# Supplementary material for: No significant boron in the hydrated mantle of most subducting slabs
Source: Nat Commun. 2018 Nov 2;9:4602. doi: 10.1038/s41467-018-07064-6 (PMC6214984; doi:10.1038/s41467-018-07064-6)
Supplement: Supplementary file 1 — Supplementary Information [file 41467_2018_7064_MOESM1_ESM.pdf]

McCaig et al Supplementary Information.

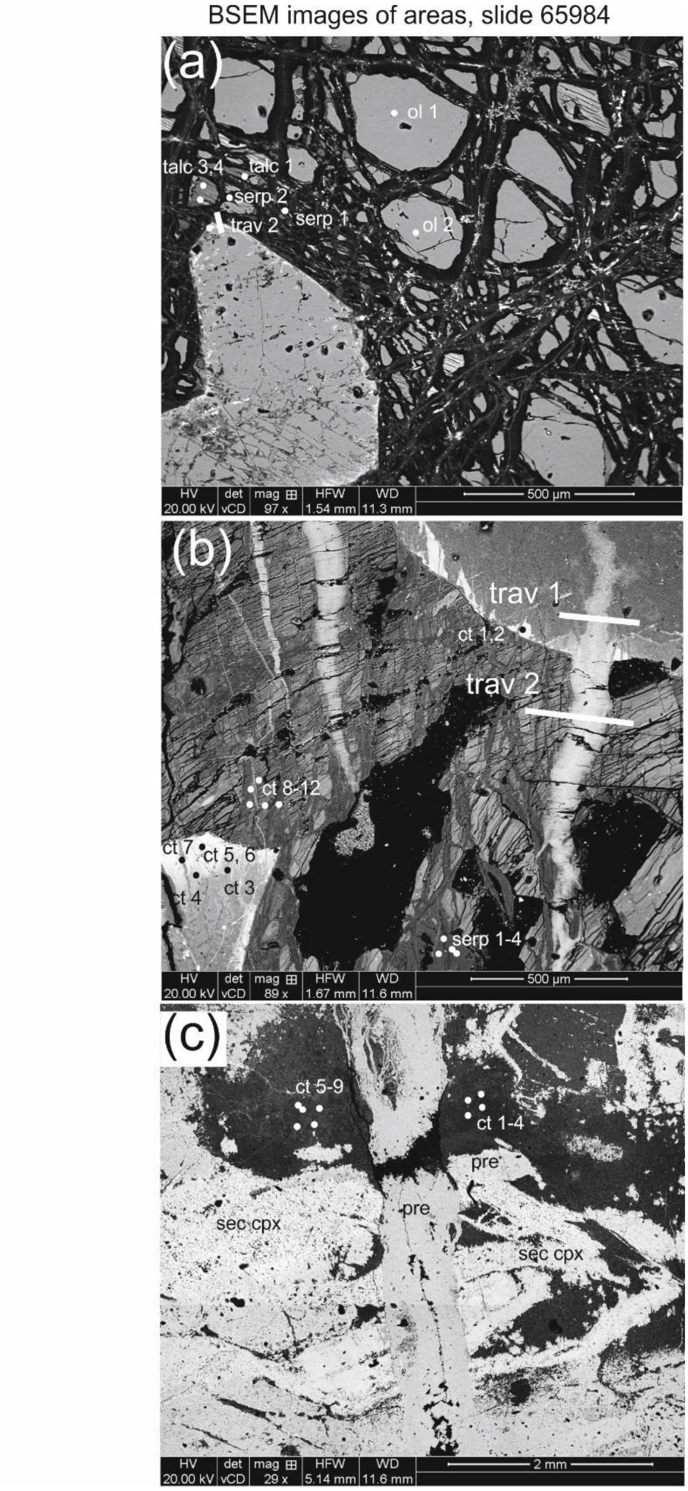

Supplementary Figure 1: slide 65984 areas and analysis points. See main text Fig. 2 for the location of areas: (a) area 1; (b) area 3; (c) area 5. Areas 2 and 4 are shown in main text Fig. 2. Pre = prehnite; cpx = clinopyroxene; ct = chlorite; serp = serpentine; ol = olive; plag = plagioclase; For analyses, see Supplementary Database 1

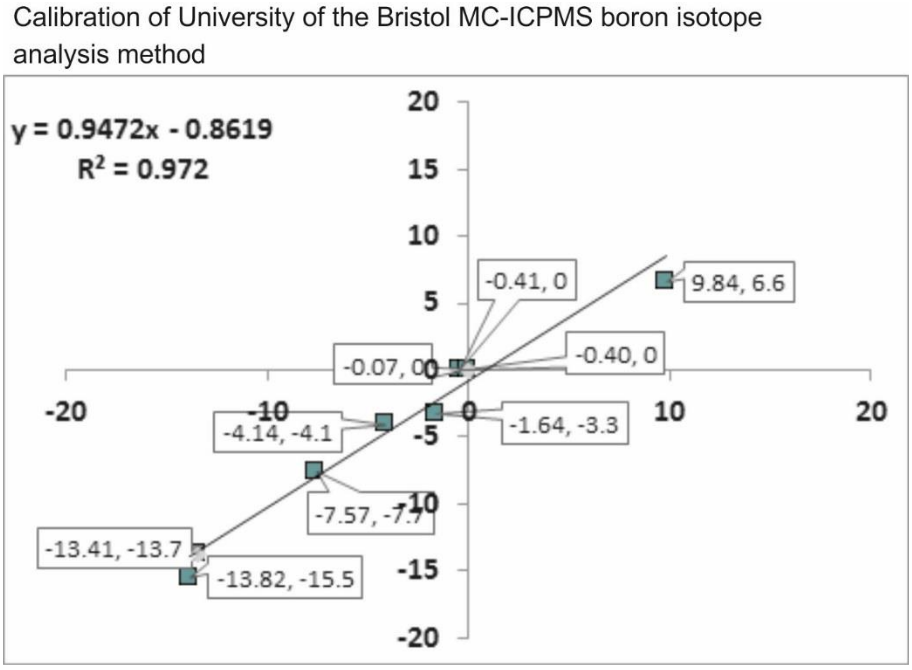

Supplementary figure 2: δ^{11}B values of boron standards widely used in the geological literature. Here samples measured in Bristol via MC-ICP-MS (x-axis) are contrasted against samples measured in IGG-CNR-Pisa via TIMS (y-axis). The specific values from the two labs are also shown in the corresponding callouts, and in table Supplementary material 2. By definition the δ^{11}B values of the boric acid SRM 951 are 0‰.

Supplementary Table 1: calibration data for standards analysed at Pisa by TIMS, and Bristol by MC-ICPMS

Univ. Bristol		CNR-IGG-Pisa	
δ^{11}B	2sd	δ^{11}B	Sample name
-.40	0.09	0	951A_008 (boric acid SRM951)
-.41	0.46	0	951B_012 (boric acid SRM951)
9.84	0.86	6.6	AF18SE_038 (IGG-Pisa in house standard)
-4.14	0.16	-4.1	B4_020 (tourmaline, Elba island)
-7.57	0.32	-7.7	B5_030 (Mt Etna basalt, erupted 22-11-1998)
-1.64	0.09	-3.3	B6_014 (Lipari obsidian, Aeolian islands)
-13.82	0.36	-15.5	PRM1A_032 (IGG-Pisa in-house standard)
-0.07	0.26	0	951S_004 (boric acid SRM 951)
-13.41	0.10	-13.7	AW_006 (serpentinite)

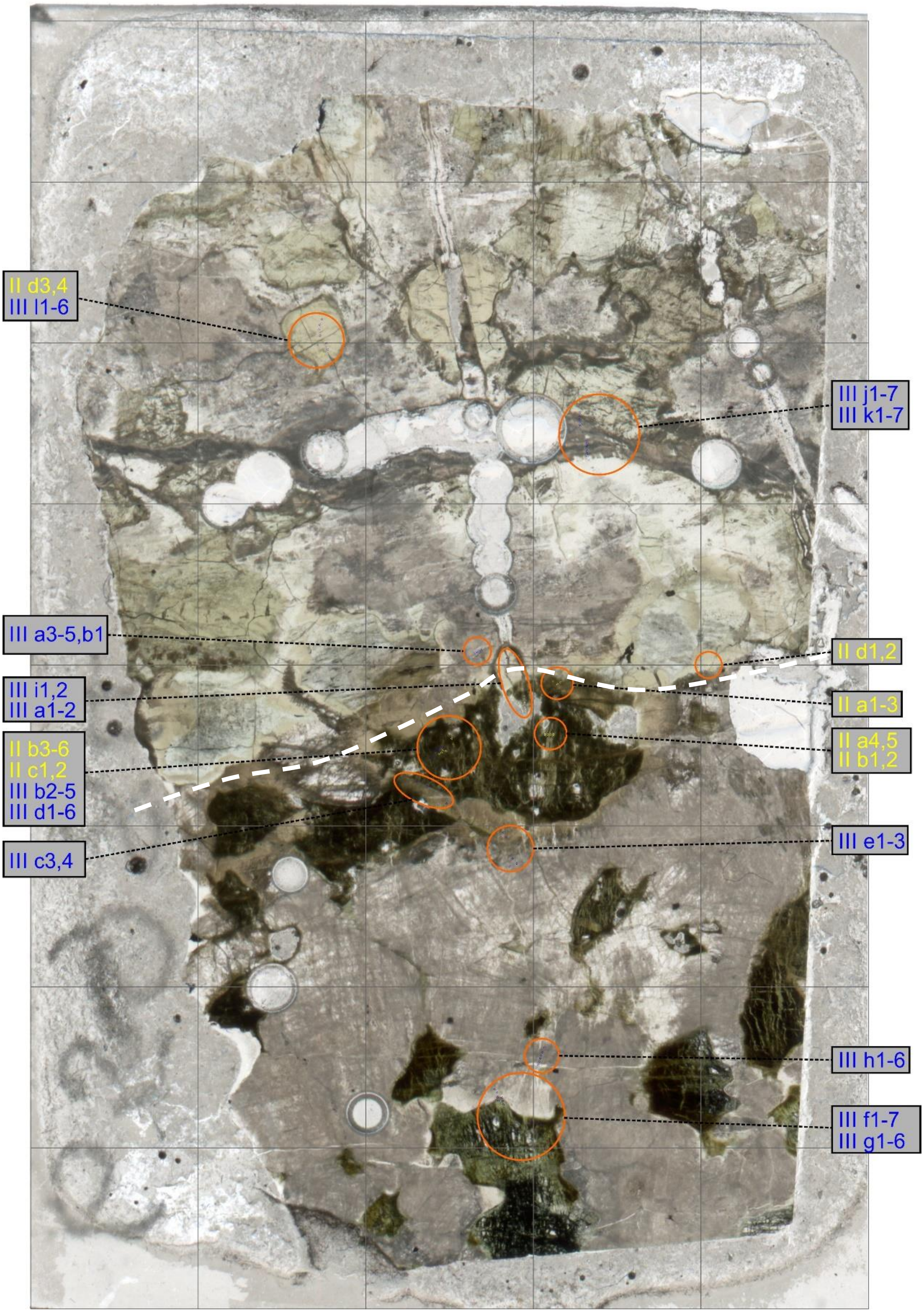

Supplementary Figure 3: Locations of LA-ICPMS analyses in Supplementary Database 2 and Fig. 3(b). This is a scan of a different thin section cut from the same block as shown in Fig. 2. White dashed line shows the boundary between background alteration (below) corresponding to AM15, and overprinting alteration (above) corresponding to AM16.

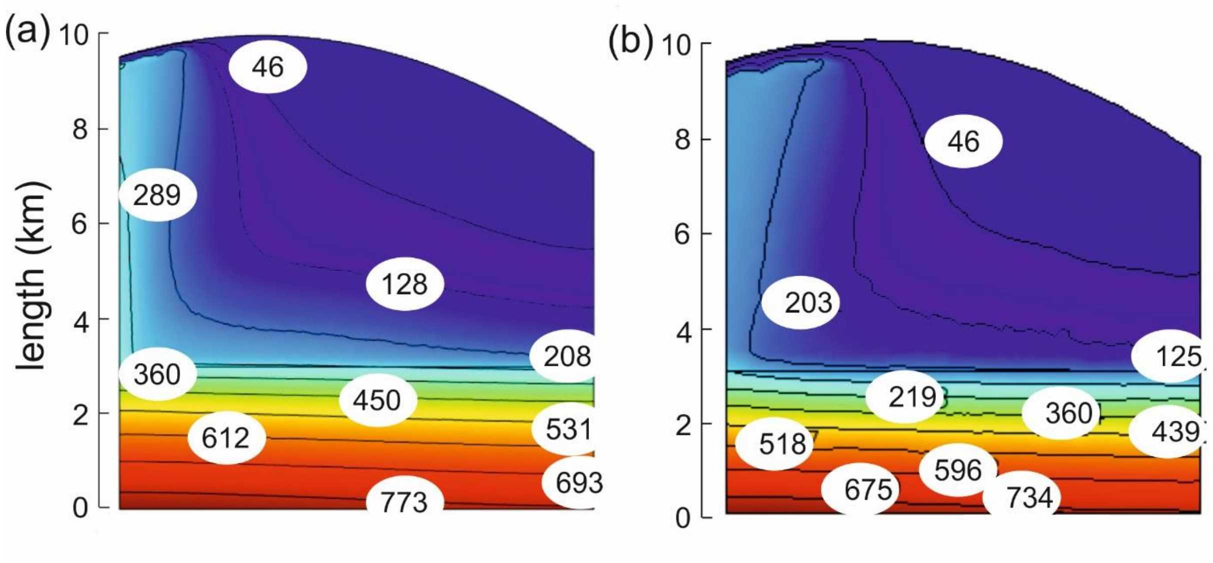

Supplementary Figure 4: Temperature snapshots (°C) of models at ~ 10^5 years. Horizontal scale = vertical. Slice shown is parallel to the fault slot (Fig. 4(a), main text). (a) 1km wide slot. (b) 2 km wide slot. This model is a continuation of the one shown in main text Fig. 4(c). Note that temperatures are higher in the narrower slot.
