# Supplementary material for: No significant boron in the hydrated mantle of most subducting slabs
Source: Nat Commun. 2018 Nov 2;9:4602. doi: 10.1038/s41467-018-07064-6 (PMC6214984; doi:10.1038/s41467-018-07064-6)
Supplement: Supplementary file 3 — Description of Additional Supplementary Files [file 41467_2018_7064_MOESM3_ESM.pdf]

Description of Additional Supplementary Files

File Name: Supplementary Data 1
Description: Electron Microprobe data contained in main text Fig. 2. Analysis points are given in main text Fig. 2 and Supplementary Fig. 1

File Name: Supplementary Data 2
Description: SiO_2 values are assigned using probe data - see methods. For analysis points see Supplementary Fig. 3

File Name: Supplementary Movie 1
Description: Temperature evolution of a model with a 1 km wide slot, with permeabilities and boundary conditions as given for Fig. 4. Time is in seconds, with a total duration of ~ 0.5 m.y. The plane of the video is parallel to the slot in Fig. 4a. Note that venting starts in the shallowest bathymetry, and then shifts to the edge of the model.
